# Cancer immunotherapy with immune checkpoint inhibitors (ICIs): potential, mechanisms of resistance, and strategies for reinvigorating T cell responsiveness when resistance is acquired

**DOI:** 10.1186/s12935-023-02902-0

**Published:** 2023-04-10

**Authors:** Hany E. Marei, Anwarul Hasan, Giacomo Pozzoli, Carlo Cenciarelli

**Affiliations:** 1 Department of Cytology and Histology, Faculty of Veterinary Medicine, Mansoura University, Mansoura, 35116 Egypt; 2 Department of Mechanical and Industrial Engineering, College of Engineering, Qatar University, Doha, Qatar; 3 Pharmacology Section, Department of Health Care Surveillance and Bioethics, Università Cattolica del Sacro Cuore, Rome, Italy; 4 Fondazione Policlinico Universitario A. Gemelli IRCCS, Rome, Italy; 5 Institute of Translational Pharmacology-CNR, Rome, Italy

**Keywords:** Immune checkpoint inhibitor (ICIs), Acquired resistance, Immunotherapy, Melanoma, Non-small cell lung cancer, Renal cell carcinoma, Breast cancer, Prostate cancer, Glioblastoma

## Abstract

Cancer is still the leading cause of death globally. The approval of the therapeutic use of monoclonal antibodies against immune checkpoint molecules, notably those that target the proteins PD-1 and PD-L1, has changed the landscape of cancer treatment. In particular, first-line PD-1/PD-L1 inhibitor drugs are increasingly common for the treatment of metastatic cancer, significantly prolonging patient survival. Despite the benefits brought by immune checkpoint inhibitors (ICIs)-based therapy, the majority of patients had their diseases worsen following a promising initial response. To increase the effectiveness of ICIs and advance our understanding of the mechanisms causing cancer resistance, it is crucial to find new, effective, and tolerable combination treatments. In this article, we addressed the potential of ICIs for the treatment of solid tumors and offer some insight into the molecular pathways behind therapeutic resistance to ICIs. We also discuss cutting-edge therapeutic methods for reactivating T-cell responsiveness after resistance has been established.

## Introduction

Immune checkpoint inhibitors (ICIs) are monoclonal antibodies (mAbs) that target inhibitory checkpoint molecules expressed by cell membrane of antigen presenting cells (APCs) and CD4^+^ T cells [1, 2]. The development of ICIs has opened a new front in the fight against several types of cancers, including but not limited to melanoma, kidney, and lung cancer, and is expected to change the current conventional interventions for diverse cancers [3].

The activated T cells, B cells, and NK (natural killer) cells can all express PD-1, a protein that belongs to the immunoglobulin superfamily [4, 5]. Since PD-1 and CTLA-4 are expressed on the surface of activated T cells, both of them are recognized as essential regulators of the delicate balance between efficient T-lymphocyte activation and over activation of T-cell functions which may result in deleterious immunopathology [6].

**Fig. 1:**
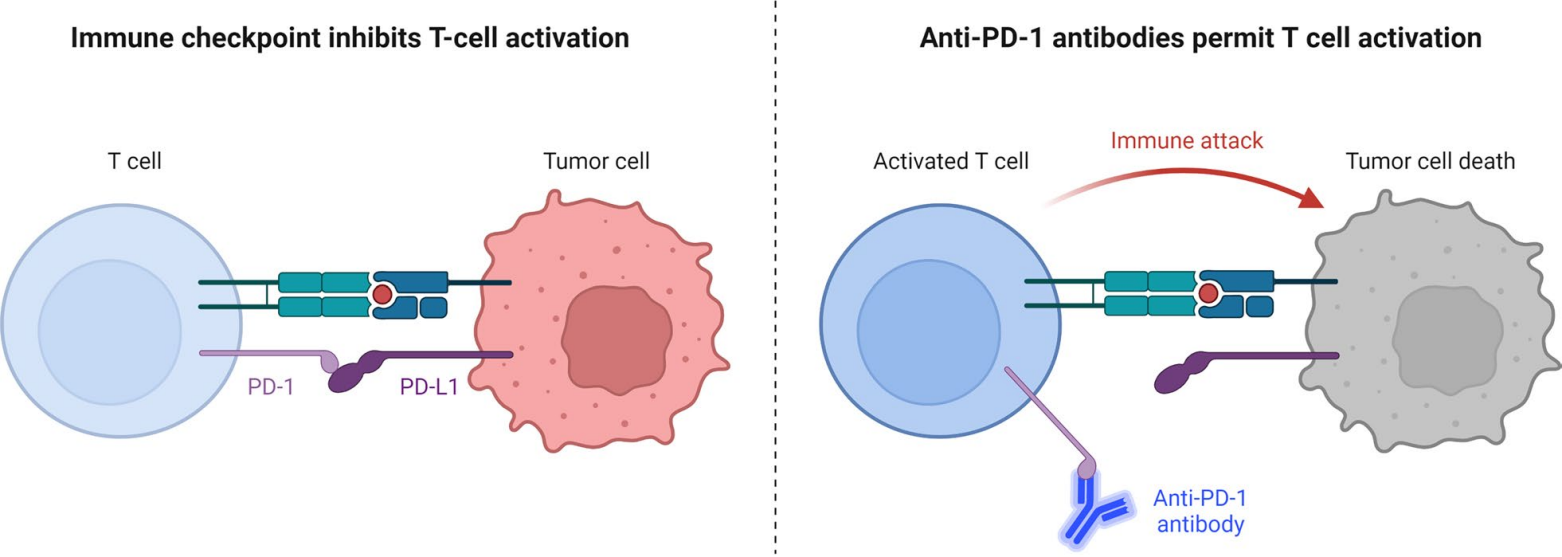
Immune Checkpoint Inhibitor against Tumor Cell. Through the interaction between PD-1 expressed on the surface of T cells and PD-L1 expressed on the surface of tumor cells, the immunological checkpoint prevents T-cell activation. Through contact between PD-1 on the surface of T cells and anti-PD-1 antibodies, T cell activation and immunological attack are enabled

**Fig. 2:**
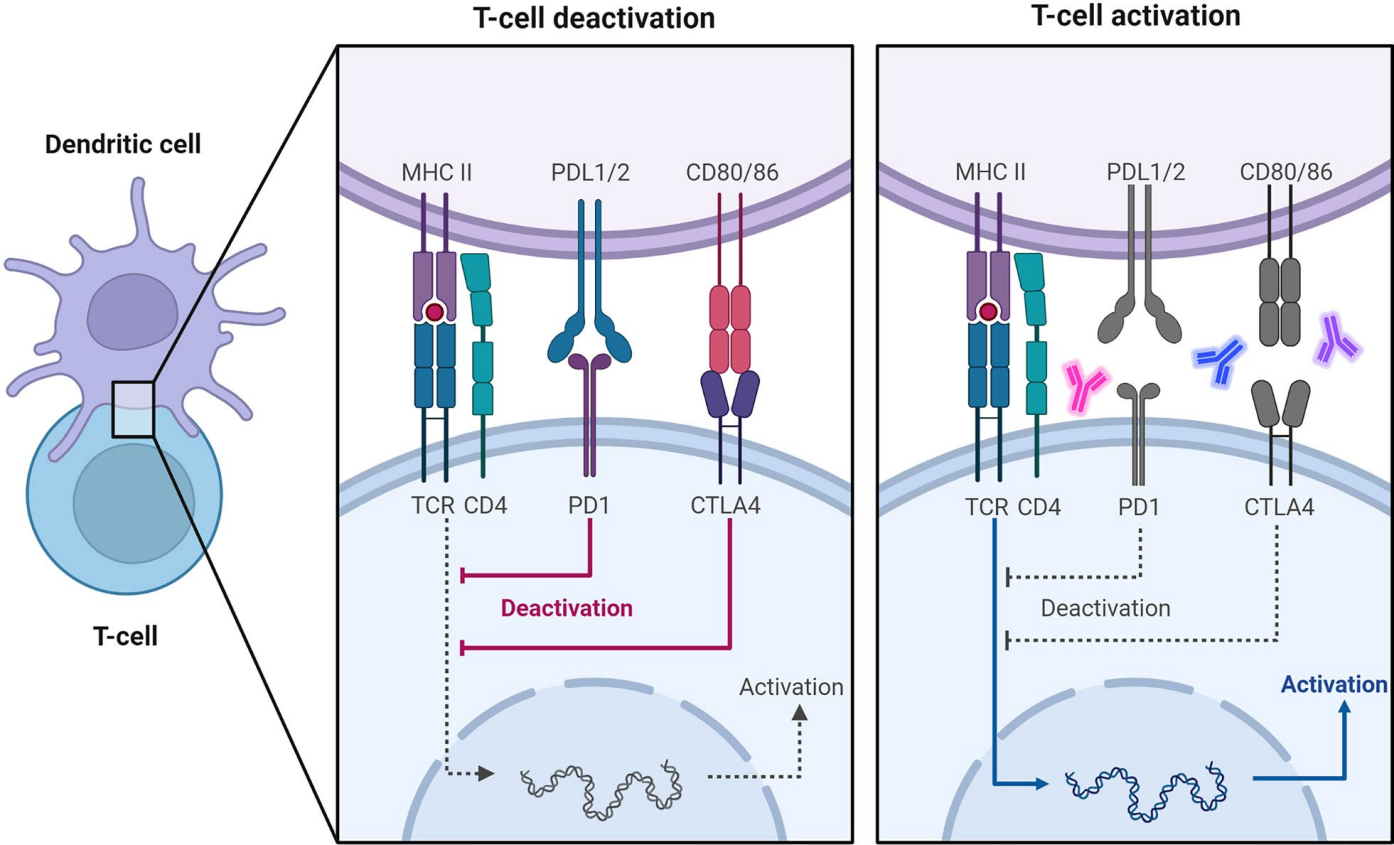
The interaction between the TCR and the tumor-specific antigen shown in the context of MHC II results in T cell activation. Following the activation of a number of downstream targets that PD-1 releases in response to interaction with either of its ligands, programmed cell death-ligand 1 (PD-L1) or 2 (PD-L2), deactivation of T cells takes place, which ultimately leads to the suppression of cytotoxic T lymphocytes (CTL). While controlling CTL activity may work as a brake to lower the likelihood of autoimmunity against host antigens, inhibiting CTL activation will be employed by forming tumor cells to evade the host's immune surveillance, which will lead to the growth of the tumor. The interaction of ICIs mAbs to PD-1, PD-L1, PD-L2, and CTLA4 restores T cell activation and slows the growth of tumors

A series of downstream targets are released by PD-1 in response to engagement with one of its ligands, programmed cell death-ligand 1 (PD-L1) or 2 (PD-L2), ultimately resulting in the inhibition of cytotoxic T lymphocytes (CTL) (Figs. 1 and 2). Halting CTL activities is seen as a double-edged sword because it can have both positive and negative consequences on the host immunological surveillance mechanisms. While regulation of CTL activity may operate as a brake to reduce the possibility of autoimmunity against host antigens, suppression of CTLs activation will be used by developing tumor cells to elude the host's immune surveillance, which will result in tumor progression [7].

By employing ICI mAbs to stop the interaction between PD-1 and its ligands (PD-L1 and PD-L2), the PD-1/PD-L1-induced immunosuppression was reversed, this in turn, revived the cytotoxic functions of CTLs against tumor antigens, leading to inhibition of neoplastic growth [8].

The engagement of CTLA-4 with its ligand (CD80/86) may also result in immunosuppression against developing tumor cells, by mediating immune evasion and escape mechanism of tumor cells [9]. Utilizing ICIs to block inhibitory PD-1/PD-L1 and CTLA-4/CD80/86 signaling pathways improves the generation of efficient immune responses against cancer cells, revitalizes tumor antigen recognition, and ultimately leads to tumor death [10]. Tim-3 (T cell immunoglobulin and mucin 3), Lag-3 (lymphocyte activation gene 3), VISTA (programmed death-1 homolog), and Tigit are other immune checkpoint molecules/targets [11].

After the FDA's 2011 approval of the CTLA-4 inhibitor (ipilimumab), six additional ICIs have received FDA clearance [1]. Of those, three (nivolumab, pembrolizumab, and cemiplimab) are PD-1 inhibitors and three (PD-L1 inhibitors) (atezolizumab, avelumab, and durvalumab). Oncologists frequently use these ICIs in their routine treatment for about 15 different tumor types, and they have demonstrated impressive success. For key parameters for use of FDA approved PD-L1 testing for ICIs, the reader can refer to Wang et al. [12]. Beginning in 2014 and continuing into 2018, the FDA approved a number of ICIs targeting PD- 1, and anti-PD-L1 drugs (Table 1). An innovative PD-1 immune checkpoint inhibitor Cemiplimab [1]. The human programmed death receptor-1 (PD-1) monoclonal antibody cemiplimab (LIBTAYO®; cemiplimab-rwlc), which binds to PD-1 and prevents it from interacting with PD-L1 and PD-L2, is being developed by Regeneron Pharmaceuticals and Sanofi Genzyme. The drug was approved in the USA in September 2018 for the treatment of patients with metastatic cutaneous squamous cell carcinoma or locally advanced cutaneous squamous cell carcinoma who are not candidates for curative surgery or curative radiation. The drug is being studied as a treatment for a variety of cancers [13].

**Table 1:** A list of active and completed immuno checkpoint inhibitor (ICI) clinical trials for various malignancies

Study name /ID	Immune checkpoint inhibitors (ICIs)	Conditions	Molecular Target	Interventions	Phase	Status	Study start date/first posted	Study completion date/last posted	Results posted
NCT03313804	Nivolumab, Atezolizumab or Pembrolizumab	Advanced Disease HNSCC	PD-1, PD-L1	Radiation therapy and ICIs	Phase 2	Recruiting	October 26, 2017	February 2025	No
NCT05329532	Pembrolizumab	HNSCC, Breast, ovarian or renal cancer	PD-1	Modi-1/Modi-1v, Pembrolizumab	Phase 1/2	Recruiting	April 7, 2022	June 30, 2026	No
NCT03544723	Nivolumab, Atezolizumab, Pembrolizumab, or durvalumab	Recurrent or Metastatic HNSCC and other Tumors Approved for Anti-PD-1 or Anti-PD-L1 Therapy (melanoma, renal, gatric, cervical cancer etc.…)	PD-1, PD-L1	Ad-p53 and Immunotherapy	Phase 2	Recruiting	October 1, 2018	December 31, 2022	No
NCT03050060	Nivolumab, Atezolizumab or Pembrolizumab	Recurrent NSCLC, advanced Melanoma, or Kidney Cancer	PI3K, PD-1, PD-L1	Hypofractionated Image Guided Radiotherapy With Concurrent Nelfinavir and ICIs	Phase 2	Terminated	February 10, 2017	June 22, 2022	No
NCT03014648	Atezolizumab	Advanced NSCLC previously treated with either nivolumab or pembrolizumab	PD-L1	ICIs	Phase 2	Terminated	July 18, 2017	March 31, 2022	No
NCT03841110	Nivolumab, pembrolizumab or atezolizumab	Advanced solid tumors (NSCLC, Breast, Gastric, pancreatic etc.…)	PD-1, PD-L1	FT500 as Monotherapy and in Combination With ICIs	Phase 1	Active not recruiting	February 15, 2019	November 17, 2022	No
NCT03693014	Ipilimumab, Nivolumab, Pembrolizumab, Atezolizumab	Lung, melanoma, bladder, renal and Head and Neck cancer	CTLA4, PD-1, PD-L1	Stereotactic body radiation therapy (SBRT) and ICIs	Phase 2	Recruiting	October 2, 2018	November 18, 2022	No
NCT03774732	Pembrolizumab	NSCLC, NSCLC metastatic	PD-1	ICI and Chemotherapy With Concurrent Irradiation	Phase 3	Recruiting	December 13, 2018	July 28, 2022	No
NCT04902040	Nivolumab, Pembrolizumab, Atezolizumab, Avelumab, Durvalumab, Plinabulin and Radiation Therapy	Advanced Melanoma, Advanced Bladder Carcinoma, Advanced Malignant Solid Neoplasm	PD-1, PD-L1	Plinabulin with ICIs and Concurrent Irradiation	Phase 1/2	Recruiting	April 14, 2021	June 1, 2025	No
NCT03115801	Atezolizumab, Nivolumab, Pembrolizumab	Metastatic Genitourinary Cancers	PD-1, PD-L1	Immunotherapy Plus Radiotherapy	Phase 2	Terminated (lack of accrual)	April 14, 2017	September 22, 2021	Si
NCT05287464	Pembrolizumab	Metastatic Renal Cell Carcinoma (mRCC)	PD-1, VEGF	pembrolizumab plus axitinib	ND	Recruiting	March 18, 2022	September 2, 2022	No
NCT05607953	Pembrolizumab	Locally Advanced Pancreatic Ductal Adenocarcinoma	PD-1, TLR9	pembrolizumab plus SD-101	Phase 1	Recruiting	November 7, 2022	November 23, 2022	No
NCT03727880	Pembrolizumab	Resectable Pancreatic Ductal Adenocarcinoma	PD-1, FAK	pembrolizumab plus defactinib	Phase 2	Recruiting	November 1, 2018	March 23, 2022	No
NCT03979066	Atezolizumab	Resectable Pancreatic Ductal Adenocarcinoma	PD-L1, HA	Atezolizumab and PEGPH20	Phase 2	Terminated	June 7, 2019	December 23, 2020	Si
NCT04666740	Pembrolizumab	Metastatic Pancreatic Ductal Adenocarcinoma	PD-1, PARP	Pembrolizumab and Olaparib	Phase 2	Recruiting	December 14, 2020	November 14, 2022	No
NCT05273554	Pembrolizumab	Advanced Pancreatic Ductal Adenocarcinoma	Pembrolizumab and VEGFR1/2/3	Pembrolizumab Plus Lenvatinib	Phase 1	Recruiting	March 10, 2022	October 14, 2022	No
NCT03952325	Pembrolizumab, Atezolizumab or Nivolumab	Triple-Negative MBC	PD-1, PD-L1, Tubulin	CPIs and Tesetaxel	Phase 2	Terminated,tesetaxel discontinued	May 16, 2019	July 30, 2021	No
NCT04638751	Pembrolizumab, Nivolumab, Ipilimumab, Atezolizumab, Durvalumab, Avelumab, Cemiplimab	TNBC, NSCLC, CRC, pancreatic cancer	PD-1, PD-L1	Immunotherapy and Chemotherapeutic Agent	Observational	Recruiting	November 20, 2020	December 2024	No
NCT03406858	Pembrolizumab	Castration-Resistant Prostate Carcinoma	PD-1, HER2	Pembrolizumab, HER2Bi-Armed Activated T Cells	Phase 2	Active, not recruiting	January 23, 2018	November 15, 2022	No
NCT03572478	Nivolumab	Prostate or Endometrial Cancer	PD-1, PARP	Nivolumab, rucaparib	Phase 1/2	It was terminated due to lack of efficacy	June 28, 2018	March 9, 2021	Si
NCT02423928	Ipilimumab	Prostate Cancer	CTLA4, DNA	Dendritic cell based cryoimmunotherapy, Cyclophosphamide, Ipilimumab	Phase 1	Completed	April 22, 2015	October 25, 2019	No
NCT02601014	Ipilimumab and Nivolumab	Metastatic Hormone-Resistant Prostate Cancer Expressing androgen receptor-variant-7 (AR-V7)	CTLA4, PD-1, Androgen hormone	Ipilimumab, Nivolumab, Enzalutamide	Phase 2	Completed	November 10, 2015	February 3, 2022	Oncotarget. 2018 Jun 19;9(47):28,561–28,571. Prostate . 2021 May;81(6):326–338
NCT03673787	Atezolizumab	Solid Tumor, Glioblastoma Multiforme and Prostate Cancer Metastatic with PI3K hyperactivation	PD-L1, AKT1	Ipatasertib, Atezolizumab	Phase 1/2	Recruiting	September 17, 2018	November 2023	No
NCT02113657	Ipilimumab	Metastatic Castration-Resistant Prostate Cancer	CTLA4	Ipilimumab	Early Phase 1	Completed	April 14, 2014	January 10, 2020	No
NCT03047473	Avelumab	Newly Diagnosed Glioblastoma Multiforme	PD-L1	Avelumab	Phase 2	Completed	February 9, 2017	July 21, 2022	No
NCT03422094	Nivolumab, Ipilimumab	Newly Diagnosed, Unmethylated Glioblastoma	PD-1, CTLA4	poly-ICLC (NeoVax), Nivolumab, Ipilimumab	Phase 1	Terminated (Manufacturer changed focus to cell therapy)	February 5, 2018	October 27, 2021	No
NCT02550249	Nivolumab	Glioblastoma	PD-1	Neoadjuvant Nivolumab	Phase 2	Completed	September 15, 2015	April 11, 2017	No
NCT03367715	Nivolumab, Ipilimumab	Newly Diagnosed, MGMT Unmethylated Glioblastom	PD-1, CTLA4	Nivolumab, Ipilimumab, short-course of Radiation Therapy (RT)	Phase 2	Completed	December 11, 2017	July 1, 2022	Si
NCT03707457	Nivolumab, Ipilimumab	First Recurrence of Glioblastoma	PD-1, GITR, IDO1, CTLA4	Nivolumab and Anti-GITR Monoclonal Antibody MK-4166. IDO1 inhibitor and INCB024360 Ipilimumab	Phase 1	Terminated	October 16, 2018	July 2, 2020	No
NCT02617589	Nivolumab	Newly-diagnosed Glioblastoma	PD-1, DNA	Nivolumab, Temozolomide, Radiotherapy	Phase 3	Completed	December 1, 2015	March 29, 2022	Neuro Oncol. 2020 Sep; 22(9): 1233–1234

Melanoma, non-small cell lung cancer (NSCLC), and glioblastoma (GBM) were among the malignancies for which ICIs were initially licensed [14, 15]. In ovarian cancer patients, a subset of patients with advanced disease and high grade tumors that express PD-L1 may be effectively treated with anti-PD-1 ICI [16]. PD-1/PD-L1 given alone have unknown effects in triple-negative breast cancer (TNBC) patients [17]. The combined use of PD1/PD-L1 inhibitors with chemotherapy significantly boosted the pathologic complete response rates (CR) in TNBC patients, according to an analysis of 4,187 patients [18]. The growing introduction of ICIs into clinical practice is often constrained due to their side effects on immune system, and rarely identified glomerular disorders [19, 20].

Due to different types of resistance to ICIs (primary or intrinsic versus secondary or acquired), most cancer patients receiving ICIs in combination with chemotherapy experience disease progression and mortality [21]. Therefore, new alternative treatment are required to enhance long-term survival in these patients, both as a preventive action and in the event that ICI-based therapy fails [22].When a patient initially responds to ICI therapy for a brief period of time before displaying symptoms of clinical or radiologic disease progression, this is referred to as acquired resistance. Patients with primary resistance do not respond to ICI treatment at all and cancer advances quite rapidly [3, 22, 23].

In this review, we will tackle the potential application of ICIs therapy in various solid tumor types. A list of putative underlying molecular pathways associated to the establishment of ICIs resistance will also be provided. Moreover, we'll take a close look at the state-of-the-art of therapeutic strategies being developed to treat ICI-resistant cancers.

### Potential of ICIs in the treatment of different cancer

ICIs have fundamentally altered how cancer is treated clinically. The percentage of patients who can benefit from ICIs is rather low, despite the fact that cancer immunotherapy has so far showed promise in a variety of cancers. Unavoidable issues include immune-related side effects and excessive expense. Hence, there is an urgent need for biomarkers that identify patients who will benefit from ICIs. One reasonable biomarker for predicting how well anti-PD1/PD-L1 immunotherapies will work is the expression of programmed cell death-ligand 1 (PD-L1). Yet, due to its variable definition, threshold, and spatial/temporal variability, its value is currently in question. Recently, it was revealed that certain gene mutations, neoantigen expression, mismatch repair status, deficient mismatch repair (dMMR), high levels of microsatellite instability (MSI-H) across the genome, "cold" vs "hot" and tumor mutational burden (TMB) may all serve as predictors of ICI treatment efficacy [24]. In Table 2 we summarized cancer types and features before diving into the individual cancer description including MSI/dMMR, "cold" vs "hot", clinical trials and outcomes/response, and approved commercial products.

**Table 2:** A summary of the cancer types, and features that affects response to ICIs

Cancer type	Features	Microsatellite instability/deficient mismatch repair	Cold vs hot	Clinical trials	Outcomes/response	Approved commercial products
Head and Neck Cancer	Squamous cell carcinoma, usually arises in the mucosal lining	Can occur in a subset of cases	Hot	Various clinical trials ongoing	Response to treatment depends on stage and other factors	Cetuximab, Pembrolizumab, Nivolumab, Durvalumab
Lung Cancer	Non-small cell carcinoma, usually arises in the lung tissue	Can occur in a subset of cases	Hot	Various clinical trials ongoing	Response to treatment depends on stage and other factors	Pembrolizumab, Nivolumab, Atezolizumab, Durvalumab
Melanoma	Malignant tumor of melanocytes, usually arises in the skin	Can occur in a subset of cases	Hot	Various clinical trials ongoing	Response to treatment depends on stage and other factors	Ipilimumab, Pembrolizumab, Nivolumab, Dabrafenib, Trametinib
Renal cell carcinoma	Arises in the cells lining the small tubes in the kidney	Can occur in a subset of cases	Cold	Various clinical trials ongoing	Response to treatment depends on stage and other factors	Nivolumab, Cabozantinib, Pazopanib, Bevacizumab
Pancreatic cancer	Arises in the cells lining the ducts of the pancreas	Usually not associated with MSI/dMMR	Cold	Various clinical trials ongoing	Response to treatment depends on stage and other factors	Abraxane, Gemcitabine, FOLFIRINOX, Onivyde, Erlotinib
Breast cancer	Arises in the breast tissue	Can occur in a subset of cases	Hot	Various clinical trials ongoing	Response to treatment depends on stage and other factors	Tamoxifen, Anastrozole, Fulvestrant, Palbociclib
Prostate cancer	Arises in the prostate gland	Usually not associated with MSI/dMMR	Cold	Various clinical trials ongoing	Response to treatment depends on stage and other factors	Leuprolide, Enzalutamide, Abiraterone, Apalutamide
Glioblastoma	Aggressive tumor of the brain, arises from glial cells	Usually not associated with MSI/dMMR	Cold	Various clinical trials ongoing	Response to treatment depends on stage and other factors	Temozolomide, Bevacizumab, Lomustine, Carmustine

### Head and neck cancer

Table 1 provides a list of few ongoing clinical trials utilizing various ICIs types against various cancer types. Head and neck squamous cell carcinoma (HNSCC) is still the sixth most prevalent cancer worldwide. More than 830,000 new cases and 430,000 fatalities are included in annual reports [25]. More than half of patients with locally advanced HNSCC relapsed despite receiving routine point-of-care therapy [26]. Two monoclonal anti-PD-1 antibodies, nivolumab and pembrolizumab, are the first ICIs approved for the treatment of recurrent HNSCC [27]. Through the PD-1/PD-L1 pathway, these immunotherapeutic drugs suppress inhibitory signals to boost the cellular immune response induced by T cells [28]. Pembrolizumab was approved for patients whose tumors are PD-L1 positive, either alone or in conjunction with chemotherapy [29].

Anti-PD-1 drugs, the current standard of therapy, have changed how HNSCC is managed with chemotherapeutic and targeted therapies [30]. Overall response is still relatively mild despite the fact that anti-PD-1 antibodies are superior to chemotherapy in relation to halting tumor development and survival [31]. The effectiveness of durvalumab for treating HNSCC, either as a single therapy or when combined with the CTLA-4 inhibitor tremelimumab, compared to chemotherapy was studied in phase II and III clinical trials [32–34]. A very small percentage of patients in clinical trials looking at ICIs for HNSCC actually benefit from treatment, according to the data, highlighting the importance of patient selection before beginning immunotherapy. Predictive biomarkers are urgently needed to enable more informed therapy selection because not all patients respond to ICIs and others may exhibit more significant tumor responses if treated with chemotherapy or other therapies. One characteristic of a tumor that can predict response to ICI therapy in a variety of cancer types is its tumor mutational burden (TMB). In a pan-cancer investigation involving more than 1600 patients, increased TMB was linked to longer survival and higher ICI therapy response rates. The optimal predictive cut-point varied greatly by histology, indicating that there is unlikely to be a single tissue-neutral definition of high TMB that is useful for predicting ICI response, despite the fact that this effect was observed in the majority of cancer types, indicating that TMB underlies fundamental aspects of immune-mediated tumor rejection. To possibly develop a tissue-agnostic predictor of effectiveness from ICIs, more thorough prediction models combining TMB with additional parameters, such as genetic, immunologic, and clinic-pathologic indicators, will be required [35].

Therefore, there is an urgent need for a fuller knowledge of immune resistance mechanisms, which are likely influenced by the action mode of ICIs. A network meta-analysis (NMA) study compared anti-PD-1 and anti-PD-L1-based therapy for HNSCC patients, proving that there are no differences that are statistically significant between the two groups [27].

### Lung cancer

ICIs can now be used in more situations without concurrent chemotherapy or targeted therapy. For NSCLC, ICIs may be utilized either as first-line or secondary treatment [36]. For NSCLC patients treated with PD-1 inhibitors as opposed to chemotherapy, 5-year overall survival (OS) rates ranged from 13 to 25% [47] in the second line and as high as 32% in the first line, according to multiple studies [37].

Validated criteria for long-term immunotherapy survival have so far demonstrated various degrees of accuracy. Although PD-L1 expression, tumor mutational burden (TMB), and interferon gamma (INFγ) are believed to be markers of response rates, these characteristics have not consistently applied to all tumor types, show a range of temporal and spatial variability, and are changeable with different types of therapy [38]. Recently, a variety of cancer types have been subjected to a more extensive evaluation of TMB, and it has been shown that this may not be a good predictor of prognosis [39]. Patients with high TMB who are not treated often have worse prognoses than individuals with low TMB, although the use of ICIs has changed this trend. Patients with non-small cell lung cancer (NSCLC) and melanoma who have higher TMB are more likely to benefit from ICIs than those who have lower TMB, according to numerous studies in particular [39]. Even so, some studies found no association between TMB and the survival of patients receiving ICIs, while others even found the opposite association [40]. Finding relevant predictive biomarkers could also be more challenging by the current propensity to combine ICIs with chemotherapy, targeted drugs, and/or other novel treatments. Numerous studies have highlighted key aspects of ICI-based survival outcomes. Extensive research covering tumor microenvironment (TME) studies, clinical surrogates, and disease mutation burden (TMB), and multi-omics data will be required to ensure the best use of ICIs and combination therapy. Overall survival (OS) for patients with metastatic disease significantly increased after the regulatory approval of PD-1 or PD-L1 inhibitors for NSCLC; currently, the majority of NSCLC patients receive PD-1/PD-L1 inhibitors as part of standard care, typically given as front-line therapy [41, 42]. Other studies showed that combinations of first-line immunotherapy, whether or not including chemotherapy, improved long-term survival, with a 4-year OS of 29% for nivolumab plus ipilimumab and a 2-year OS of 38% for nivolumab plus ipilimumab and chemotherapy [43, 44]. Despite the successful use of ICIs over the past decade in patients with NSCLC, lung cancer remains the most common cause of cancer mortality worldwide [45].

### Melanoma

Skin cancer known as melanoma it is well known for having a relatively low survival rate and is caused by the misregulated growth of abnormal melanocytes [46]. Interferon therapy and chemotherapy are ineffective against melanoma, however, relatively recent research into the molecular basis of the disease resulted in novel therapeutic approaches: ICIs and targeted therapies. The first drug for the treatment of melanoma to receive FDA approval was ipilimumab, a CTLA-4 inhibitor. Pembrolizumab was authorized to treat metastatic melanoma just three years later [47]. Nivolumab was the third ICI to get worldwide approval in the same year (Jin et al. 2023).

After 5–10 years of treatment, only 22% of melanoma patients exhibited clinical benefit with ipilimumab, whereas 40–45% of patients with melanoma showed positive efficacy from PD-1 inhibitor therapy. Combination with PD-1 and CTLA-4 inhibitors was more successful than treatment with either drug alone. Consequently, the risks occasionally outweigh the benefits [48].

It was reported that a combination therapy using ICIs had a 5-year OS rate of 52% [49]. Even while ICIs greatly boosted melanoma patients' chances of survival, 40–65 percent of those taking PD-1 inhibitors and more than 70 percent of those taking CTLA-4 inhibitors did not exhibit positive response, primarily because of the emergence of resistance [50]. Additionally, one third of patients that initially had positive clinical outcomes, subsequently developed tumors and acquired drug resistance [51].

### Renal cell carcinoma

The treatment of metastatic clear cell renal cell carcinoma (ccRCC) is immunotherapy-based [52].

Examples of broad strategies are ICIs and tyrosine kinase inhibitors (TKIs) that target the vascular endothelial growth factor receptor (VEGFR). Dual ICIs such as ipilimumab and nivolumab (IO/IO) are also exploited for treatment of ccRCC [66–68]. Several phase III clinical studies using TKI/IO regimens have reported objective response rates (ORRs) of 58–71% despite the fact that follow-up time is still insufficient to determine whether durable responses would be observed [53–56]. In contrast, ipilimumab and nivolumab had a 41% ORR, but nearly almost 50% of responders had responses lasting more than 4 years [57]. The best regimen for a given patient is unknown because these regimens have not been properly compared. Furthermore, it is not known if second-line TKI therapy can prolong survival in patients who fail to respond to IO/IO as a first line of treatment. Patients treated with the TKI/IO regimen and ipilimumab/nivolumab had equivalent 12-mo PFS and OS [58]. In cases of metastatic ccRCC after second-line therapy, there was no appreciable difference in PFS between patients receiving ipilimumab plus nivolumab and those receiving TKIs and ICIs [59].

### Pancreatic cancer

Pancreatic ductal adenocarcinoma (PDAC) still has a limited role in immunotherapy. According to reports, PDAC has an immunosuppressive TME and a low TMB, both of which pose challenges for pancreatic cancer immunotherapy [60]. For PDAC patients with microsatellite instability or mismatch repair deficiency (MSI-H/dMMR) who had metastatic or incurable disease, pembrolizumab was approved by FDA in May 2017 [61]. The FDA approval was based on the findings of five clinical trials that evaluated pembrolizumab in patients with incurable solid tumors who had received two prior lines of therapy. Out of 149 MSI-H/dMMR cancer patients studied over the course of the five studies, 59 responded, with an objective response rate (ORR) of 36.9% and a complete response rate (CR) of 7% [76–78]. The study by Le et al. reported in eight out of the 86 participants in the trial, the ORR was 62%.

Pembrolizumab was evaluated in a non-randomized, open-label fashion across many centers and cohorts. In 233 patients with 27 different tumor types, the ORR was 34.4%. Despite these positive pooled response rates to pembrolizumab in patients with MSI-H/dMMR cancer who had previously received treatment, the response rate in the subset of patients with MSI-H/dMMR pancreatic tumors was not as high. In the pancreatic cancer subgroup, the median OS was 4.0 months, but the median time to response was 13.4 months. It is difficult to extrapolate these results from a small number of patients with MSI-H/dMMR pancreatic cancers because the rate of mismatch repair deficiency in PDAC has been shown to range from 0.8 to 2% [62–65].

**Fig. 3:**
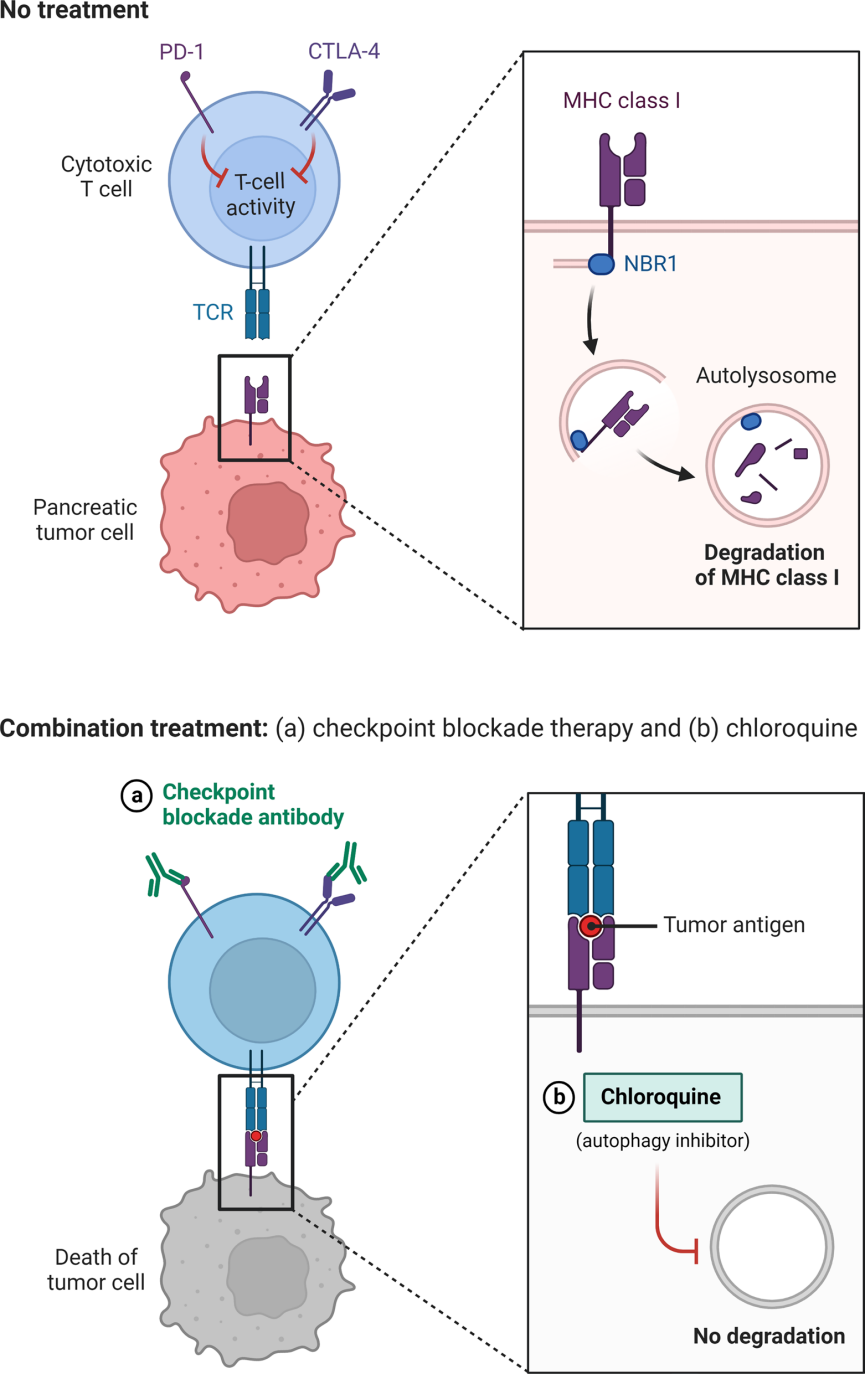
Blockade of CTLA-4 or PD-1 Signaling in Tumor Immunotherapy. Dendritic cells (DC) and naive T cells interact in the lymph node during the priming phase. The interaction between the TCR and the tumor-associated antigen shown in the context of MHC II constitutes the activation signals. The interaction between CD28 and B7 expressed on the surface of DC is one of the additional activation signals. The immune system attacks and eliminates tumor cells as a result of the effector phase, which takes place in the peripheral tissue. At this stage, PD-1 and PD-L1 inhibitory signals on T cells are suppressed, effectively activating T cells against the tumor antigen

Long considered to be an immunologically "cold" cancer, PDAC has a number of factors that make it difficult for immunotherapy to be effective [66]. The logical next step is to try to overcome these obstacles by combining anti-PD-1/anti-PD-L1 checkpoint inhibitors with other immunological and targeted therapy (Fig. 3).

In the first phase II randomized clinical study investigating dual immune checkpoint therapy with anti-PD-L1 antibody durvalumab with or without anti-CTLA-4 antibody tremelimumab, O'Reilly et al. observed unfavourable outcomes in patients with advanced PDAC [67]. Study participants had previously received a chemotherapy regimen based on fluorouracil or gemcitabine. The ORR was 3.1% for individuals who received durvalumab and tremelimumab together. Effective MSI-H/dMMR immunotherapy for PDAC continues to be elusive, and clinical research in this area is ongoing. Studies combining PD-1/PD-L1 suppression with other PDAC therapies are presently under progress [68].

### Breast cancer

Breast cancer can currently be categorized into three primary subtypes based on the expression of the estrogen and progesterone receptors (ER and PR) and HER2 (also known as ERBB2): luminal ER positive and PR positive (further divided into luminal A and B), HER2 positive, and triple-negative breast cancer (TNBC) [69, 70]. Breast cancer has recently surpassed lung cancer as the most common type of cancer in the world, with an estimated 2.3 million new cases annually, or 11.7% of all cancer cases [45].

TNBC and HER2 breast cancer subtypes displayed increased tumor biomarker expression levels in response to immunotherapy, as well as improved immune infiltration and immunogenicity [71], 72]. Treatment with PD-1 checkpoint inhibitors showed good efficacy against these subtypes. For instance, the ORR to atezolizumab therapy for TNBC is 25%, while for pembrolizumab therapy for ER + in tamoxifen-taking patients is 4% [69].

Immunotherapy has several disadvantages when utilized to treat breast cancer due to its significant heterogeneity. Despite this, PD-1/PD-L1 inhibitors can still increase the T cell infiltrate in patients' TME when used in conjunction with other therapies. This prevents tumor immune escape and increases the anti-tumor effects of PD-1/PD-L1 inhibitors [73, 74].

Thirty-two of the 111 patients with metastatic TNBC who tested positive for PD-L1 expression had received weekly intravenous pembrolizumab 10 mg/kg. The median PFS was 1.9 months. One patient (3.7%) experienced complete remission (CR), four (14.8%) experienced partial remission (PR), and seven (24.9%) experienced stable status [75, 76].

Pembrolizumab is administered intravenously every two weeks in a phase II clinical trial to two cohorts of patients with metastatic TNBC: (A) an unselected population of advanced patients, and (B) a first-line cohort of PD-L1-positive tumors. The 170 patients in cohort A had a median OS of 9.0 months, a median PFS of 2.0 months, and an ORR of 5.7% for PD-L1 positive patients [77]. The median PFS, median OS, and ORR for the 84 patients in group B were 2.1 months, 18.0 months, and 21.4%, respectively [78]. The importance of PD-1/PD-L1 inhibitors in early therapy is demonstrated by the fact that various treatment regimens would dramatically impact the response rate of PD-L1-positive patients. For metastatic TNBC, PD-1/PD-L1 inhibitors in combination with other immunotherapies have demonstrated some promising therapeutic effects, such as the ability of atezolizumab and nab-paclitaxel to change patient prognosis. A number of targeted therapies (including radiotherapy, oncolytic virus therapy, CDK4/6 inhibitors, MEK inhibitors, AKT inhibitors, and vaccines) are combined with PD-1/PD-L1 inhibitors. Clinical prognosis improvements for people with metastatic TNBC are currently possible [17].

### Prostate cancer

The second most common cancer in the world is prostate cancer (PCa) [79]. Radical prostatectomy or radiation therapy may be used to treat localized PCa, nonetheless, the outlook for advanced or metastatic PCa is dismal [80].

Recent studies have demonstrated excellent responses to ICIs and/or their combinations regimen in a subset of patients with high levels of PD-L1 expression in the tumor, CDK12 mutations, high levels of TMB, or high levels of microsatellite instability (MSI), and low levels of mismatch repair (dMMR). Therefore, to improve the management of this condition, immunotherapy remains a desirable therapeutic choice for prostate cancer and not only [81]. As far as PCa concerns, in a phase I trial, two of fourteen patients with metastatic castration-resistant prostate cancer (mCRPC) showed PSA declines of ≥ 50% after receiving a single intravenous dose of ipilimumab, and the drug was accepted well [82].

In a different Phase I trial using tremelimumab (a humanised anti-CTLA-4 antibody) and androgen deprivation using bicalutamide for recurrent prostate cancer, in three out of eleven patients, the PSA doubling time was prolonged [83].

Ipilimumab was given to 50 patients in a phase I/II research for those with metastatic CRPC (mCRPC), or in combination with radiation. Six patients had stable illness, one had a full response, and eight had PSA decreases of less than 50% [84].

In a phase 3 trial, radiation was followed by ipilimumab or a placebo for 799 individuals with mCRPC [85]. The OS between the ipilimumab and placebo groups did not differ statistically, nevertheless. Instead, PFS showed a statistically significant increase [86, 87].

Ipilimumab and nivolumab (anti-PD-1) were administered in combination to patients with mCRPC in a phase II clinical trial, which achieved a 25% ORR and was associated with substantial adverse effects [88]. Atezolizumab, avelumab, and durvalumab given alone or in combination regimen are additional treatment options for mCRPC [89]. Since prostate cancer exhibits multiple immunosuppressive characteristics associated with low TMB, low expression of PD-L1, and sparse T-cell infiltration, it has been referred to as an immunologically "cool" tumor. Nevertheless, for some people with prostate cancer, immunotherapy is still a viable option. High MSI/dMMR or CDK12 mutations in prostate cancer may make them more sensitive to ICIs in clinical settings [90].

### Glioblastoma

Anti-PD-1/PD-L1 therapy for glioblastoma (GBM) has been shown to be both safe and effective in GBM mice models. Longer life times and a considerable reduction in tumor mass size has been observed. Clinical trials including patients with recurrent GBM are now testing PD-L1 [91]. Despite the fact that ICIs are effective against a number of cancers, the majority of glioblastoma (GBM) patients do not react to ICI therapy [92]. Clinical trials in phase 2/3 have not yet proven that the administration of PD-1 inhibitors to patients with GBM significantly improves overall survival (OS), either when combined with other treatments or when used alone currently considered to be standard of care [93]. Newly diagnosed O6-methylguanine-DNA-methyltransferase (MGMT) methylated GBM, nivolumab was given to upfront radiation and temozolomide in CheckMate 298 [94].

Recurrent GBM patients received either nivolumab or bevacizumab, and the results showed that bevacizumab had a longer PFS of 3.5 months compared to nivolumab and no difference in OS [95]. In a phase 2, there was an advantage in PFS from combination therapy of 4.1 months over pembrolizumab alone [96]. Recurrent GBM patients who received nivolumab together with either bevacizumab (10 mg/kg) or bevacizumab (3 mg/kg) every two weeks showed no change in PFS or OS [97].

Durvalumab’s effects alone or plus radiotherapy in GBM patients [98], or bevacizumab-refractory recurrent GBM have been disappointing. Despite these unpleasant results, there is still more to discover. A small group of GBM patients may benefit from ICI therapy, as evidenced by the fact that the median duration of response for the few nivolumab responders (7.8%) was statistically longer than the bevacizumab cohort (11.1 months versus 5.3 months) [95].

Cloughesy et al. discovered that neoadjuvant anti-PD-1 (pembrolizumab) therapy enhanced CD8 + T cell infiltrate and INFγ-related gene expression in the tumor of recurrent GBM [99]. Instead, a paucity of T cells but a significant infiltration of immunosuppressive CD68 + macrophages were discovered by De Groot et al. in patients' tumor tissue who had already received treatment, which may play a role in the emergence of resistance to anti-PD-1 therapy [100].

The blood–brain barrier (BBB), which will be covered in more depth below, makes treating GBM significantly harder than treating other solid tumors. The BBB creates a selectively permeable barrier across the majority of central nervous system (CNS) blood arteries in order to separate the tumors from therapeutic access [101]. Due to aberrant neovasculature and irregular blood flow, GBM also has a so-called blood–brain tumor barrier (BBTB), which affects the therapy of the tumor when medications are administered systemically and further prevents pharmaceuticals from leaving the circulation [101]. While creating a treatment for GBM, there are a few approaches to get around the BBB. The first step is to create a treatment that is better able to cross the BBB's endothelial cells. An agent's ability to create hydrogen bonds, polarity, or lipophilicity can all be decreased to achieve this [102]. Using the "Trojan horse" technique is a second way to get around the BBB [102]. In this technique, a substance that is typically incapable of entering the brain is coupled to a monoclonal antibody that is directed against one of the BBB's transcytosis receptors. The chemical can enter the brain undetected because the endothelial cell is prompted to permit entry by the binding of the monoclonal antibody to the receptor. Moreover, there has been some success in using nanotechnology to deliver treatments across the BBB. For instance, a medicinal substance can pass through the BBB and infiltrate the tumor when it is carried by a liposome containing an antibody that targets transferrin [103]. A therapeutic drug can be delivered to the brain using inorganic nanoparticles (IONPs) with an iron oxide core while also serving as an imaging agent for MRI. This enables the tracking of the delivery of therapeutic agents to the tumor itself [103]. Other studies have shown that certain peptides can be conjugated with therapeutic molecules to deliver treatment directly to the tumor while sparing the surrounding brain from damage, allowing these peptides to cross through the BBB and home to the tumor [104]. Radiation, electroporation, low intensity ultrasound, among other methods, can physically damage the BBB, allowing medicines to enter the brain [105]. Low-intensity pulsed ultrasound (LIPU) was utilised in a recent Phase I/IIa clinical experiment to damage the BBB and let a medication enter the brain. During the trial, patients had the SonoCloud-1 device implanted into their skull bones so that pulsed sonication could be administered. The research revealed that patients tolerated LIPU well and that carboplatin could penetrate the brain after being sonicated [46]. In a canine model, irreversible electroporation (IRE) has been demonstrated to break the BBB and eradicate tumor cells [106]. The IRE system's testing revealed some shortcomings that this technology has been updated to address; this improved technique is known as high-frequency irreversible electroporation (H-FIRE). Convection enhanced delivery (CED) also avoids the BBB and delivers medication directly to the tumor or to an area around it. ICIs will be better able to target the immunological checkpoint receptors that are expressed in the GBM tumor microenvironment and enable more effector cells to enter the tumor by creating therapies that are more suited to passing through the BBB, disrupting the BBB, or bypassing the BBB entirely. The delivery and concentration of ICI, which interacts with the tumor and may enhance treatment outcomes in GBM patients, could be more precisely controlled with the use of CED.

### Mechanisms of ICIs resistance

ICIs have fundamentally changed how cancer is treated for many different tumor types, giving some patients a level of survival that was previously unattainable. However, despite many patients first responding favorably to ICIs, they frequently acquired resistance with time. The effective development of next-generation immunotherapies may be hampered by a lack of understanding of the processes driving acquired resistance to ICIs. The response rate of PD-1 inhibition in many diseases (including melanoma, Merkel cell carcinoma, Hodgkin's lymphoma, and MSI high malignancies) ranges from 40 to 70% in unselected patients [107]. Unfortunately, the majority of other recognized advanced cancers such as advanced non-small-cell lung cancer, advanced or metastatic urothelial cancer (mUC) and advanced renal-cell carcinoma only have response rates of 10% to 25% [108–110]. In conclusion, only a small percentage of them actually achieve the long-lasting response.

In contrast to acquired resistance, which typically describes patients who initially respond to therapy for a while before eventually experiencing clinical and/or radiologic disease progression, primary resistance typically describes patients who don't react at all and instead progress quickly or eventually with ICIs. In order to fight the issue of primary resistance, a significant amount of work has gone into designing combination approaches, typically with empiric complementary drugs. For instance, ICIs, multi-target TKIs, and EGFR inhibitors have all been used in conjunction with chemotherapy in the treatment of lung, breast, stomach, and renal cell carcinoma [111]. Additionally, a systematic search for biomarkers that can anticipate the initial ICI response has been conducted. PD-L1 expression, tumor mutational burden, and tumor infiltrating lymphocytes (TILs) have all been explored as potential predictors, and numerous more markers are now being investigated [112]. On the other hand, there haven't been any approved pharmacological advancements for preventing or reversing acquired resistance.

### Clinical evidence and molecular mechanisms associated with acquired resistance to ICIs

Neoantigen-specific T cells may have a significant impact on how the body reacts to ICIs. Recently, it has been demonstrated that the long-term effect of ICIs blockage in NSCLC and melanoma correlates with somatic mutational and neoantigen density. Epigenetic suppression, immunological evasion, and clinical advancement may coexist with the absence of somatic mutations encoding putative tumor-specific neoantigens [113]. The absence of many neoantigen-specific T lymphocytes raises the possibility that pressure selection to eradicate these clones led to the development of acquired resistance [114]. No loss-of-function mutations in HLA genes such as B2M, JAK1 or JAK2 were found in a clinical study of 4 patients with NSCLC who had acquired resistance to ICIs (170). Exome analysis of pre vs post-treatment tissue, however, revealed deletion of a large number of mutations that were expected computationally to develop into neoantigens at the time resistance was developed.

**Fig. 4:**
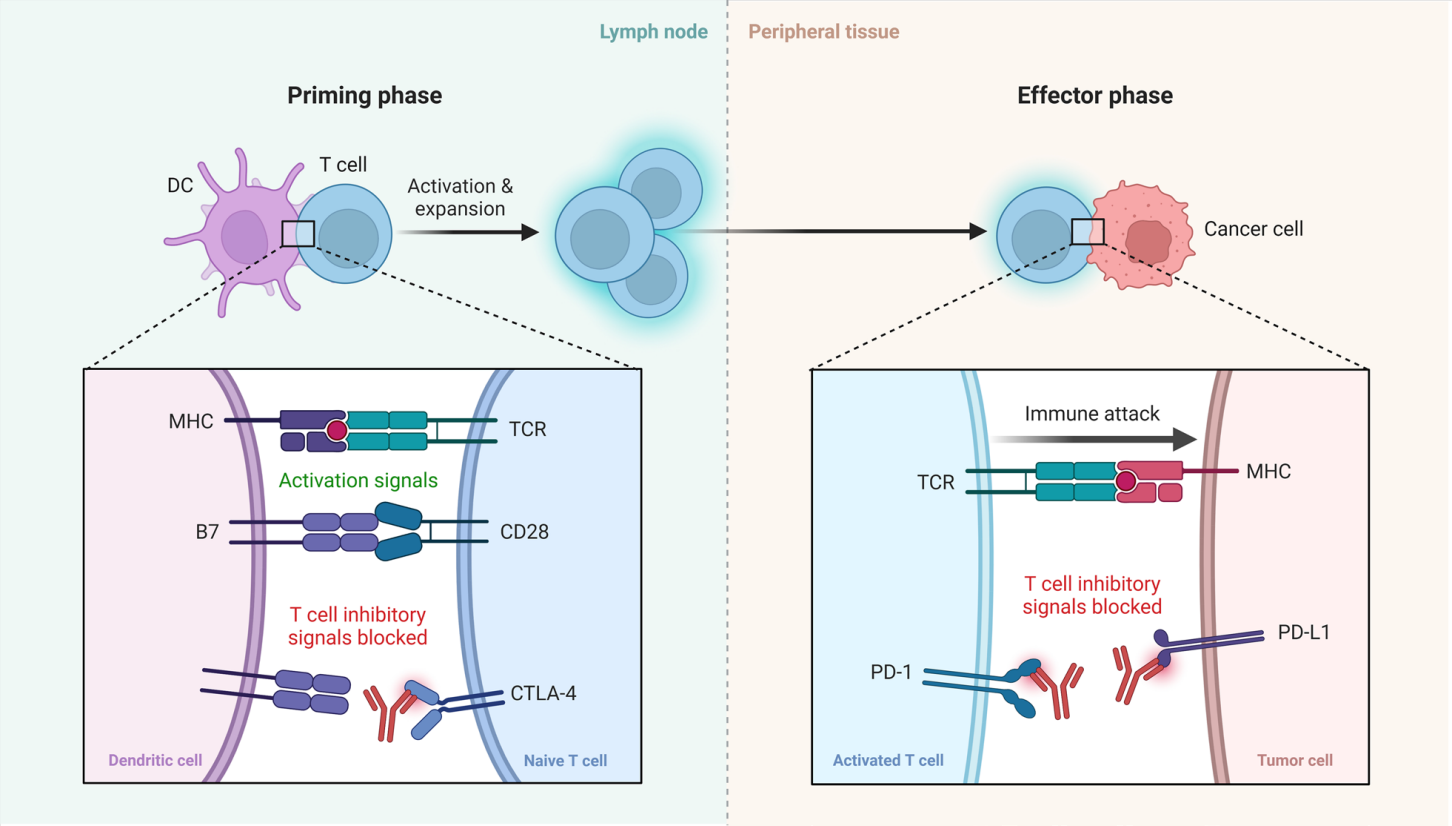
Combination Treatment of Pancreatic Cancer. In PDAC, the autophagy cargo receptor NBR1 directs an autophagy-dependent pathway that targets MHC-I molecules for lysosomal degradation. MHC-I is more frequently identified inside autophagosomes and lysosomes than on the cell surfaces of PDAC cells. Notably, restoring surface levels of MHC-I in syngeneic host mice results in improved antigen presentation, increased anti-tumor T cell responses, and inhibition of tumor growth. To enhance the anti-tumor immune response, dual ICI therapy (anti-PD1 and anti-CTLA4 antibodies) is used in conjunction with autophagy suppression, either genetically or pharmacologically with chloroquine

Antigen-presenting cells' Major Histocompatibility Complexes (MHCs) are required for the activation of T-cell mediated immunity (Fig. 4). The coordinated expression of several genes is what allows MHC class I to deliver tumor antigens [115–117].

The beta-2-microglobulin (B2M) gene is required for both the stability of the MHC class 1 molecule at the cell surface and the facilitation of peptide loading (173). Loss of function mutations in B2M have previously been demonstrated to cause MHC I loss and serve as a biological pathway for tumor escape from immune detection [118].

One of the common findings in acquired resistance to ICIs has recently been identified as truncating changes in B2M. Using data from 4 melanoma patients who had developed immunotherapy resistance. One patient was found to have a homozygous acquired truncating B2M mutation, according to Zaretsky et al. [119]. In melanoma and other tumor forms, acquired defects in antigen presentation are observed. One patient exhibited B2M loss of heterozygosity (LOH) and two frameshift mutations, and another melanoma patient had two frameshifts B2M changes at the time of disease progression [120]. B2M changes were shown to be more prevalent in non-responders to anti-CTLA4 therapy [121].

In several studies of acquired resistance to immune checkpoints inhibitors in lung cancer, and MMR-d cancers were described homozygous deletion of B2M and alterations of B2M respectively [24]. Additionally, 4 of 9 patients and 3 of 9 patients, respectively, had considerably lower levels of the B2M protein and the MHC class 1 protein without any corresponding B2M molecular changes, according to Gettinger et al. [122]. Other unknown genomic or nongenomic variables may change MHC class 1 expression and affect resistance to ICIs under these circumstances (Fig. 4).

### Role of IFN signaling in ICIs resistance

The JAK-STAT pathway, which regulates the expression of MHC class I and PD-L1 in tumor cells, is activated by the release of IFNγ from effector T cells. This signalling chain reaction may result in tumor cell death [123]. Both the JAK1 (JAK1 Q503*) and JAK2 genes have acquired loss-of-function mutations in two individuals who had side effects with ICIs after 1 and 2 years (JAK2 F547 splice-site mutation) [119].

Increased surface HLA class 1 and PD-L1 expression as well as considerable IFNγ pathway activation are seen in patients with JAK1/2 heterozygous mutations [124].

Genomics modifications in JAK1, JAK2, or IFNGR1 has been linked to primary immunotherapy resistance [125]. Ipilimumab, a medication that targets the CTLA-4 protein, was ineffective against melanomas that had deletions (copy number loss) of crucial IFNγ pathway genes including IRF1, IFT1/2, and amplifications (copy number increase) of IFNγ-related pathway inhibitors like SOCS1 and PIAS4 [125]. It is unclear how much other IFNγ pathway chromosomal aberrations besides JAK1 and JAK2 affect the development of acquired resistance to ICIs, and there are few clinical reports on the acquisition of these alterations.

### Immunosuppression/exclusion caused by tumors

Loss of the tumor suppressor PTEN raises the expression of immunosuppressive cytokines and lowers T-cell effectors, which limits T-cell-driven infiltration and immunity. PTEN is crucial for regulating PI3K activity in preclinical models [126]. A patient with metastatic uterine leiomyosarcoma who had previously shown a virtually full response to pembrolizumab for more than 2 years exhibited PTEN deletion, according to a recent study [127]. Similarly, melanoma patients who had acquired resistance to immune therapies, reported PTEN loss [23]. The generation of immunosuppressive cytokines, modifications in dendritic cell priming, activation of regulatory T cells, and a lack of significant T cell infiltration in melanoma have all been associated with Wnt/-catenin pathway activity [128].

### Other inhibitory checkpoints

When resistance is gained, the expression of TIM3, LAG3, and V-domain Ig suppressor of T cell activation (VISTA) increases, however it is unclear whether these modifications are directly linked to resistance [129]. Such checkpoints may occasionally be linked to T cell depletion and terminal malfunction, but in other contexts they may also be linked to T cell activation (Blank et al., 2019). Even with many of the research mentioned above, it can be challenging to confirm or identify a specific resistance mechanism. VISTA is a type I transmembrane protein. In particular for triple-negative breast cancer, VISTA is a potential immunological therapeutic target because to its association with immunotherapy resistance. It is found in regulatory T cells and myeloid-derived suppressor cells in large concentrations, and functional inhibition of it is proven to slow tumor growth [130]. It is still unclear how common acquired resistance to ICIs actually is because some authors deduce the resistance mechanism from circumstantial evidence.

### Therapeutic strategies for disrupting acquired resistance

Several therapeutic strategies that target one or more of the major biological pathways, including the IFNγ pathway, other immunological checkpoints, the tumor microenvironment, and epigenetic modification, have been developed to combat acquired resistance to ICIs.

Numerous clinical trials focusing on JAK1/2 and STAT are currently being conducted. In a phase 1/2 research, advanced NSCLC patients received either osimertinib alone or in conjunction with the JAK1-selective inhibitor AZD4205 (NCT03450330). SC-43, a SHP-1 agonist that inhibits STAT3, is undergoing a phase 1/2 clinical trial for NSCLC when combined with cisplatin (NCT04733521).

The stimulation of IFN genes (STING) showed an increase in anti-tumor immunity through the production of proinflammatory chemokines and cytokines, including type I IFNs [131]. STING agonists like E7766, GSK3745417, and MIW815 are now undergoing clinical studies (NCT04144140, NCT03843359, and NCT03172936, respectively).

Patients with PD-L1 + NSCLC were enrolled in the phase 2 clinical study (CITYSCAPE) to compare the anti-TIGIT antibody tiragolumab with atezolizumab versus placebo plus atezolizumab. Overall response rates improved (Rodriguez-Abreu et al., 2020). Additional drugs that target the tumor microenvironment have been explored such as inhibitors of CSF1R, TGFβ, VEGF, IL-1/6, A2AR, CD73, IDO1, and B7-H4. DNA methylation and histone alterations are examples of epigenetic changes [132]. The enzyme DNA methyltransferase (DNMT), which controls the silence of genes and non-coding genomic regions, mediates DNA methylation. Histone modification enzymes like histone methyltransferase (HMT) and histone deacetylase alter the structure of chromatin, which affects how genes are regulated (HDAC) (Kanwal and Gupta, 2012). Immunotherapy resistance may be treated with epigenetic modification enzyme inhibitors, such as DNA methyltransferase inhibitors (DNMTis), histone methyltransferase inhibitors (HMTis), and histone deacetylase inhibitors (HDACis) [133].

According to preclinical research, HDACi and DNMTi both improve the responsiveness to anti-PD-1 therapy in a variety of malignancies [134]. Enhancer of zeste homolog 2 (EZH2), one of the histone methyltransferase enzymes, is associated to the expansion, migration, and invasion of malignant cells, such as glioblastoma, ovarian, and prostate cancer. Inhibiting EZH2 along with anti-CTLA-4 and IL-2 immunotherapy had silencing effects on antigen presentation and immune response [135].

The PD-1/PD-L1 pathway is not the only mechanism slowing down antitumor immunity in the majority of cancer patients, and inhibiting the PD-1/PD-L1 axis does not enough stimulate an efficient antitumor immune response. Certain combinations of treatments, such as -PD-1/PD-L1 plus radiotherapy, chemotherapy, angiogenesis inhibitors, targeted therapy, other immune checkpoint inhibitors, agonists of the co-stimulatory molecule, stimulators of interferon genes, faecal microbiota transplantation, epigenetic modulators, or metabolic modulators, have been shown to have superior antitumor efficacies and higher response rates. Moreover, -PD-1/PD-L1 moiety-containing bifunctional or bispecific antibodies also induced stronger antitumor activity. These combination techniques eliminate immunosuppressive brakes, promote numerous cancer-immunity cycle activities at once, and manipulate an immunosupportive tumor microenvironment. We outlined the synergistic antitumor efficacies and mechanisms of -PD-1/PD-L1 in this review when used in conjunction with other treatments [136].

### Future perspective

Eventually, immunotherapy took a while to break through a wall of active cancer medications. In the past ten years, ICI have been developed and approved at an extraordinary rate for a number of cancer types. ICI has made great strides, yet the problem of cancer treatment remains. Immune-checkpoint immunotherapy has unlocked a door, but the case is still open. In the coming ten years, we want to identify pharmacodynamics characteristics and biomarkers for ICI efficacy and toxicity prediction in order to optimize ICI regimens and develop novel combinations.

## Conclusions

Clinical research for the next generation of immunotherapies for patients with primary and acquired resistance is ongoing despite the lack of notable results. A deeper comprehension of the underlying biology may allow for more specific application of immunotherapies other than immune checkpoint inhibitors, leading to more effective therapeutic choices. The development of drugs and cellular therapies to prevent, avoid, or overcome ICI resistance will eventually be made possible by this advancement. In order to provide cancer patients a variety of therapeutic options, it is critical to understand the mechanisms underlying acquired resistance. In particular, the activation of the IFNγ pathway, inhibition of TGFβ, and co-suppression of immunological checkpoints like TIGIT have attracted interest as fascinating potential therapeutic strategies and are awaiting results.

## Data Availability

All data are included in the manuscript.
